# A Case of Cervical Abscess

**Published:** 1883-12

**Authors:** F. R. Campbell


### Clinical Reports.

### A Case of Cervical Abscess.

By F. R. Campbell, M. D.

The importance of the early incision of deep abscesses of the neck has been demonstrated in an able paper written by the late Dr. Lidell, of New York, and published in the *American Journal of the Medical Sciences* for October. In ten cases that he has collected, there were five deaths and five recoveries. Three of these deaths were due to asphyxia caused by œdema glottidis, one was due to septicæmia and one to hemorrhage. Of the five fatal cases, none were artificially opened, while of the five resulting favorably, all were opened before extensive burrowing had taken place, thus proving that these abscesses do not tend spontaneously to recovery. A case of cervical abscess occurred in my own practice which will serve to confirm the views taken by Dr. Lidell:

John C., aged 20, a lead worker, first noticed a hard lump, the size of a hickory nut, under angle of left jaw, October 18th. The swelling occasioned but little inconvenience, and he continued to work as usual until October 22d, when his throat became so sore that he was obliged to stay at home. The patient was seen by me October 25th, one week after the swelling was first observed. There was an indurated lump about two inches long in the submaxillary triangle, with considerable swelling of the surrounding tissues. No fluctuation could be detected. Was unable to examine fauces, as the patient could not open his mouth. Temperature, 102°. Ordered hot flaxseed poultices, frequently renewed, and, as his tongue was coated and bowels constipated, gave calomel, gr. v, to be followed by Sal Rochelle.

October 26th, swelling increased, temperature 103°. A sharp-pointed bistourie was introduced into swelling, but only a trace of pus was discovered, and none flowed afterward when the opening was enlarged with a probe. Patient was taking, at this time, tinct. ferri chlorid., ♏ xx, every three hours.

October 27th, swelling increased, left eye nearly closed; there was a diffuse cellulitis, resembling erysipelas, extending from temple down to chest. Temperature, 104½°. Respiration 28, with considerable dyspnœa. Gave quinine, gr. xij, followed by five-grain doses of the same drug every four hours, alternating with the iron. Dr. Chas. Gay was called in consultation and made an incision where pus had been previously discovered by the aspirating needle. A small amount of sanious matter was evacuated, having an intensely fetid odor, but the swelling was not perceptibly reduced. A drainage tube was inserted and the opening washed out frequently with a hot solution of carbolic acid, 1 to 40.

October 30th, pus flowing freely; temperature 102°, and swelling reduced. Suppuration continued until November 3d. At this time a probe could be passed in the opening to mesial line of neck anteriorly and backward as far as the angle of the jaw. The patient continued to improve, and has made a good recovery.

From the symptoms attending this case it seems evident that a fatal termination would have rapidly ensued if the swelling had not been incised; and the importance of opening even before well-marked collections of pus can be detected is demonstrated.

